# Intelligent Framework for Early Detection of Severe Pediatric Diseases from Mild Symptoms

**DOI:** 10.3390/diagnostics13203204

**Published:** 2023-10-13

**Authors:** Zelal Shearah, Zahid Ullah, Bahjat Fakieh

**Affiliations:** Department of Information Systems, Faculty of Computing and Information Technology, King Abdulaziz University, Jeddah 21589, Saudi Arabia; zasultan@kau.edu.sa (Z.U.); bfakieh@kau.edu.sa (B.F.)

**Keywords:** pediatric diseases, mild symptoms, recurrent symptoms, severe cases, children’s death, long-term morbidity

## Abstract

Children’s health is one of the most significant fields in medicine. Most diseases that result in children’s death or long-term morbidity are caused by preventable and treatable etiologies, and they appear in the child at the early stages as mild symptoms. This research aims to develop a machine learning (ML) framework to detect the severity of disease in children. The proposed framework helps in discriminating children’s urgent/severe conditions and notifying parents whether a child needs to visit the emergency room immediately or not. The model considers several variables to detect the severity of cases, which are the symptoms, risk factors (e.g., age), and the child’s medical history. The framework is implemented by using nine ML methods. The results achieved show the high performance of the proposed framework in identifying serious pediatric diseases, where decision tree and random forest outperformed the other methods with an accuracy rate of 94%. This shows the reliability of the proposed framework to be used as a pediatric decision-making system for detecting serious pediatric illnesses. The results are promising when compared to recent state-of-the-art studies. The main contribution of this research is to propose a framework that is viable for use by parents when their child suffers from any commonly developed symptoms.

## 1. Introduction

Machine learning (ML) techniques have been utilized in several health and medical services [1]. In medicine, machine learning is the use of machine learning models to search medical data and uncover insights to help improve health outcomes and patient experiences and improve patient care [2]. ML aids in deriving benefits from the data generated for an individual patient and through the collective experience of many patients [2]. ML models lead to improving healthcare quality and outcomes at both the patient and health facility levels [1]. They are widely used in the medical fields for several purposes, including diagnosis, directing severe medical conditions, and triage of serious cases [3].

Pediatric diseases are among the most prevalent and treatable diseases that can lead to serious long-term effects or death [4]. According to the World Health Organization statistics for 2021, about 5.2 million children died, mostly from preventable and treatable causes [5]. A UNICEF report in 2020 showed that about 6.3 million children in the previous year died from health complications [6]. Roughly 13,800 child deaths occur every day that are preventable [7]. About 25% of annual child deaths are caused by diseases that first appear as mild symptoms, such as a cough, fever, or diarrhea [8]. Globally, some common pediatric diseases, such as pneumonia, diarrhea, and malaria, remain leading causes of children’s deaths [9] despite considerable progress in the medical field. Thus, improving child survival remains a matter of urgent concern [6].

Some mild pediatric illnesses, if they are not detected or treated early, can be fatal or lead to long-term effects or prolonged complications [10]. The mild symptoms that frequently occur in childhood can be signs of severe disease, but it is difficult for parents to determine whether they signify dangerous or common diseases [6]. Even if the child’s symptoms vanish rapidly each time, it can be worrisome if they keep coming back [6]. In addition, even mild symptoms can be possible signs of serious diseases that, if not discovered and treated in time, will cause death or lead to chronic disease or long-term health issues [11]. For instance, up to 81% of child deaths occur outside a hospital due to serious and dangerous diseases that sometimes appear in a child as mild symptoms, such as pneumonia [12]. That is because the parents of the ill children did not realize the real health condition of their child, so they did not bring the child to the hospital in the early stages [9]. There are many factors that could affect early diagnosis and medical interventions for pediatric diseases, including the patient’s disease and the parent’s awareness [9].

The World Health Organization (WHO) emphasizes the importance of critical pediatric illness and urges help in any medical or technical ways to improve the early recognition of children who have severe cases and need immediate care and hospitalization [5]. Sometimes, critical illness and urgent pediatric diseases appear in children as mild or frequent symptoms [13]. If children’s health problems are not identified and treated in the early stages, they can affect the children’s mental, physical, behavioral, and emotional development [14,15]. When children’s symptoms are clearly serious, it is common for parents to observe them and bring the child to the hospital to access proper care [11]. However, children have a high risk of morbidity and mortality if they do not receive proper healthcare at the right time [6].

Parents have the difficult job of trying to make a judgment of their children’s health; it is often hard to know what health concerns are normal and what symptoms are critical or emergency signals [16]. Therefore, it will be helpful to apply ML to aid parents in recognizing severe children’s symptoms earlier [16]. Thus, this research proposes a machine learning (ML) framework to aid in recognizing serious children’s diseases that do not appear clearly in children as serious diseases ad instead show up as mild or recurrent symptoms. The proposed ML framework will aid sick children’s parents in detecting the severity of the symptoms earlier, which will enable rapid and appropriate healthcare action. The following are the study’s main contributions:

- A severity detection framework is proposed to be viable for use by parents to detect the child’s emergency case from mild symptoms in the early stage.
- A dataset was collected from real cases by pediatricians at a maternity and children’s hospital.
- Various preprocessing steps were applied to prepare and balance the dataset for training the proposed model.
- The proposed model was developed from nine machine learning (ML) methods to produce the most accurate results.
- We evaluated the proposed model for its accuracy, precision, sensitivity/recall, F1 score, and ROC AUC.
- The comparison between the nine proposed models is presented. Also, the comparison of the best-proposed model with related works is presented.

## 2. Related Works

The review of recent studies presented in this part shows that applying ML methods in medical care, like for detecting the severity of diseases, is usually faster and more accurate compared to traditional methods [15]. Also, ML aids in early detection and effective treatment, and consequently, in reducing death rates [11]. Furthermore, it can assist primary medical decision-making, as it can incorporate a significant portion of clinical data that are often ignored (or not collected at all) by clinicians, such as the recurrence of mild symptoms in a short time period; ML can offer benefits from analyzing these data instead of underestimating them [11].

### 2.1. Some of the Current Attempts to Use ML for Early Detection of Disease Severity

Effective early disease detection methods or solutions have a high priority worldwide [17]. Early disease detection research attracts more attention than the treatment of diseases and recovery or rehabilitation research [17]. Detecting severe illness in the early stages aids in preventing the progression, development, and consequent complications of disease. Especially in early childhood, early detection of severe diseases has great outcomes since many pediatric diseases can harm the child but are preventable and fully curable if treated early [18]. Therefore, public health research supports solutions that enhance medicine for early diagnosis and prevention [14]. One of the WHO’s core policies is “essential public health operation 5 (EPHO5)” [19], which concerns disease prevention, including early detection of illness. In this regard, it is important to pay attention to children’s occasional symptoms, which may relate to severe diseases [11]. Early detection is considered a type of prevention activity [20]. Researchers have proposed using several AI and ML technologies in various medical practices to achieve this WHO goal, such as for assessing the risk of disease onset and detecting the severity of symptoms [21].

In recent research, a predictive tool was proposed to identify patients with severe COVID-19 [22]. The results of the study indicated that the random forest (RF) algorithm is the most useful algorithm to predict the severity of COVID-19 cases and may facilitate effective care and further optimize resources. An ML model was proposed to triage and assess severe COVID-19 cases [23]. It was found that supervised learning had better results than unsupervised learning algorithms, with 92.9% testing accuracy [23].

### 2.2. Some of the Recent Research That Uses ML to Detect Pediatric Diseases’ Severity Earlier

Several research studies have aimed to improve pediatrics by detecting sensitive childhood disease problems early using ML models. However, most of these researchers were customizing their model for specific purposes of use or specific diseases. Iheme et al. [24] implemented a pediatric diagnostic system to aid in efficient early diagnosis of pediatric diseases, which contributes to reducing childhood mortality. The system was built with naïve Bayes and a decision stump tree based on 581 records. It was found that naïve Bayes algorithms gave high-accuracy results.

Bertsimas et al. [25] proposed a model to discover cases of children’s severe traumatic brain injury, as well as to determine children at very low risk of clinically traumatic brain injury, which helped to reduce the number of patients whose clinically important symptoms were missed.

Masino et al. [26] evaluated six different ML algorithms to identify children with severe cases of sepsis at an early stage, before clinical recognition. It was found that all ML algorithms that were used could detect sepsis before clinical signs occurred in the child with an area under the curve (AUC) between 0.80 and 0.82, with no significant differences between the results and accuracy of the different algorithms.

Mossotto et al. [27] proposed a model to classify pediatric inflammatory bowel disease. The model was able to discriminate some symptoms, but it was uncertain in discriminating other common symptoms.

Pan et al. [28] built ML models to detect Mycoplasma pneumoniae (MPP) early and rapidly in children. These models were based on logistic regression (LR), decision tree (DT), gradient-boosted decision tree (GBDT), support vector machine (SVM), and multilayer perceptron (MLP) algorithms. It was found that the most efficient results were obtained using GBDT, with the best performance and an accuracy of 93.7%.

Roquette et al. [10] developed predictive models for detection of the severity of pediatric diseases. They used two algorithms, a gradient-boosting classifier and a deep neural network (DNN), to extract information from textual data in a dataset consisting of 499,853 pediatric cases. However, this system was designed to cover just some common childhood diseases. The model achieved an accuracy of 89%.

Hwang et al. [16] built a random forest model to predict critical illness and hospitalization among children visiting the ER. The data covered 2,621,710 children’s cases. The ML model effectively predicted the children needing hospitalization, with an accuracy of 94%.

Table 1 summarizes the features of research based on ML algorithms. These researchers proposed models aimed at enhancing pediatric medicine and tried to find a solution for some common pediatric issues.

This literature review finds that ML models have succeeded in detecting some severe diseases at an early stage with optimal results. Whilst there are advantages of these systems, they have some limitations. Although different technologies were used in those solutions and had benefits, there is still a need to find solutions that can make decisions without requiring intervention by medical staff, to be usable by parents to detect severe diseases early. Therefore, there is a need to use technology in this area to enhance current solutions. Since AI and ML solutions can make a huge contribution in this area and are used widely and successfully in assessing the risk and severity of diseases [29], they could be used to cover existing gaps and aid in reducing the high rate of childhood mortality and morbidity. This research aims to enhance pediatric medicine and extend the limits of existing ML solutions. Accordingly, this research proposes a framework that harnesses the advantages of using ML algorithms and avoids the existing disadvantages.

## 3. Methods

This section will discuss the research framework and the methodology of this study.

### 3.1. Research Framework

**Figure 1:**
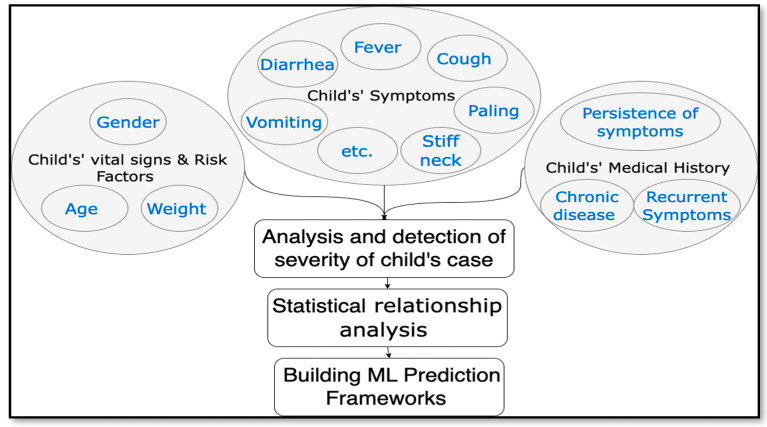
Research framework.

The purpose of the research framework is to show the research variables and to clarify relationships among the variables with regard to the research problem [30]. Major elements that are included in the research framework should represent major concepts and variables of the research [30]. The variables involved in this research were identified through the literature review and previous related research, interaction with specialist pediatric physicians, and reviewing WHO criteria for children with severe disease. In this research framework, the features that children’s disease severity depends on are categorized into four groups: the child’s vital signs, medical history, risk factors, and symptoms. Symptoms are mentioned only briefly in Figure 1 due to space limitations; however, they are illustrated in detail in Table 2 in the data overview section. The major variables of this research are shown in Figure 1.

### 3.2. Data Collection

This quantitative study used a primary dataset. The data were real cases of children’s disease, with their associated severity level, collected from outpatient children’s records, children’s emergency visits, and cases from the intensive care department (ICU) in the maternity and children’s hospital in Makkah, Saudi Arabia. The main criterion for selecting the chosen cases was that they had common and mild symptoms, but these symptoms are potentially related to mild or severe children’s diseases.

**Figure 2:**
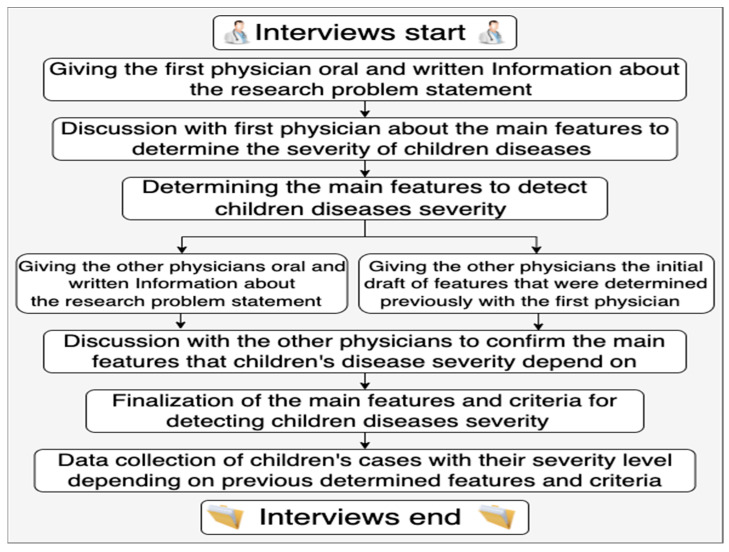
Steps of interview process.

First, interviews were conducted to determine the main features needed to detect children’s disease severity from mild/recurrent symptoms. The interviews followed a semi-structured, in-depth format with specialist and consultant pediatric physicians. An in-depth interview is a one-to-one qualitative research method that involves conducting intensive individual interviews with a small number of respondents to obtain data on a certain topic [31]. The in-depth interview is a popular data collection method in qualitative research to collect medical data [31]. In-depth interviews can be unstructured, structured, or semi-structured, but semi-structured is the most common [32]. The main advantages of using in-depth interviews are to obtain more accurate and more detailed data than are obtainable through other data collection methods, such as surveys. However, the main challenges of this method are that it takes a lot of effort and time, as the collected data must be transcribed, organized, and analyzed in detail [31]. Intelligent verbatim transcription was applied following the interviews, to capture, transcribe, and analyze the data. Intelligent verbatim transcription means writing down the responses of the interviewees minus redundant words or sounds. It is a common style of transcription known as clean transcripts, and it collects the data that can be used by the researcher for a particular purpose [32]. Then, to structure the dataset, after determining the main variables to distinguish whether the children’s cases were mild or severe, a table was developed in MS Excel using selected features to associate children’s cases with their severity level. Physicians who were involved in the process of determining the main variables performed the record review process of data from electronic health records (EHRs) for pediatric cases, and then inserted different de-identified data from children’s cases with their severity levels, which were known as either mild or severe cases. The steps of conducting the interviews are shown in Figure 2. The data were collected under ethical approval to use for research purposes.

This research also investigated some related research [4] and reviewed WHO data (ICD code 10 is an acronym used in the medical field and stands for International Classification of Diseases, tenth revision [33]) to validate the correctness of features and severity detection criteria obtained through interacting with physicians. According to the WHO, childhood extends from newborn (i.e., day 1) to 16 years old, which was appropriate for this study [34]. The essential variables/features needed to detect the severity of children’s diseases are vital signs (gender, age, and weight), children’s disease risk factors that affect the disease severity (e.g., young age), children’s symptoms, and previous health history.

### 3.3. Dataset Descriptions

Severe cases refer to cases that need urgent medical intervention to avoid long-term complications or death [35]; mild cases refer to cases that do not need urgent medical care and have no long-term effect if they are not treated immediately [35].

Common symptoms (usual symptoms) are symptoms (signs of illness or that something is wrong with the child’s body) that occur in large numbers among children [4]. Mild symptoms are symptoms that are not very strong or do not obviously seem to be severe, and patients with mild symptoms can return to normal quickly [36]. According to the WHO, and after interaction with pediatric physicians, there are 25 symptoms that are mild and common in children but that may be signs of severe cases or may relate to mild diseases. These symptoms are shown in Table 2 below.

The dataset consisted of 33 columns and 579 rows. Each row related to one child’s case. The first 32 columns in the dataset represented features that were needed to detect the children’s case severity. These features could be categorized into three main groups, which were the child’s vital signs (gender, age, weight, and body temp.), symptoms that the child had, and their medical history (chronic disease, persistence of symptoms). The last column represented the severity level of the case (target feature). These features are described in Table 2.

**Table 2:** Feature descriptions.

Feature	Datatype	Description
Gender	Binary	The child’s gender, either boy or girl. In the dataset, 0 means girl and 1 means boy.
Age (Month)	Int64	The child’s age by month, where 0 means the child’s age is less than one month.
Weight (kg)	Float64	The child’s weight in kilograms.
Body Temp.	Float64	A temporary rise in body temperature, considered a fever when at or above 37.5 °C.
Inability to drink or suckle	Binary	The child’s difficulty drinking/suckling, where 0 means the child did not have it and 1 means the child had it.
Vomiting	Binary	The forceful ejection of the stomach contents, where 0 means the child did not have it and 1 means the child had it.
Drug ingestion	Binary	The child has taken a drug not prescribed to them, where 0 means the child did not do it and 1 means the child did it.
Paling	Binary	Lack of color in the face, where 0 means the child did not have it and 1 means the child had it.
Cough	Binary	Pushing air out using chest and abdomen muscles, where 0 means the child did not do it and 1 means the child did it.
Ear pain or discharge	Binary	Ear pain or drainage of blood, ear wax, or fluid from the ear, where 0 means the child did not have it and 1 means the child had it.
Liquid or soft defecating	Binary	The advanced stage of diarrhea, where 0 means the child did not have it and 1 means the child had it.
Purulent eyes	Binary	The child’s eyes producing mucus or pus, where 0 means the child did not have it and 1 means the child had it.
Restlessness	Binary	The child being unwilling or unable to stay still or to be quiet and calm, where 0 means the child did not have it and 1 means the child had it.
Red spots (rash)	Binary	Tiny spots of bleeding under the skin, where 0 means the child did not have it and 1 means the child had it.
Diaper rash	Binary	A rash in the diaper area, where 0 means the child did not have it and 1 means the child had it.
Turbidity on the cornea	Binary	Cloudiness of the child’s eye lenses, where 0 means the child did not have it, and 1 means the child had it.
Red eyes	Binary	Red, irritated, and bloodshot eyes, where 0 means the child did not have these and 1 means the child had these.
Mouth ulcer	Binary	A sore that develops in the soft tissue lining of the gums, tongue, inner cheeks, lips, and palate, where 0 means the child did not have it and 1 means the child had it.
Constipation	Binary	Being unable to completely empty the bowels, where 0 means the child did not have it and 1 means the child had it.
Stiff neck	Binary	Stiffness in the neck muscles, where 0 means the child did not have it and 1 means the child had it.
Diarrhea	Binary	The body’s solid waste being more liquid than usual, where 0 means the child did not have it and 1 means the child had it.
Blood in feces	Binary	Bleeding in the body’s waste, where 0 means the child did not have it and 1 means the child had it.
Hollowed eyes (sunken eyes)	Binary	Sunken eyes, where 0 means the child did not have these and 1 means the child had these.
Poor abdominal skin turgor	Binary	The skin being slow to return to normal during a check (when gently grasping the skin), where 0 means the child did not have it and 1 means the child had it.
Abnormal thirst	Binary	Feeling thirsty even when the child drinks a lot, where 0 means the child did not have it and 1 means the child had it.
Fussiness/irritability	Binary	Being difficult to please or showing an unnecessary amount of care or worry about something that is not important, where 0 means the child did not have it and 1 means the child had it.
Abdominal pain	Binary	Pain anywhere between the chest and groin, often referred to as the stomach region or belly, where 0 means the child did not have it and 1 means the child had it.
Nausea	Binary	An urge to vomit, where 0 means the child did not have it and 1 means the child had it.
Falling	Binary	The child having fallen recently, where 0 means the child did not and 1 means the child did.
Persistence of symptoms	Binary	If any of the previous symptoms continued for a long time [37], where 0 means there were no persistent symptoms and 1 means one or more symptoms were persistent.
Recurrence of symptoms	Binary	Return of disease symptoms after a period of healing [13], where 0 means there were no recurrent symptoms and 1 means there were one or more recurrent symptoms.
Chronic disease (CD)	Binary	A disease that was persistent or long-lasting in its effects (common CDs in children include asthma, cystic fibrosis, diabetes, epilepsy, and developmental disabilities [38]), where 0 means the child did not have any CDs and 1 means the child had one or more CDs.
Severity (target feature)	Binary	The severity level of the associated child case, where 0 means the case was mild and 1 means the case was severe.

### 3.4. Data Cleaning and Preprocessing

The collected raw data usually cannot be used directly in performing the analysis process. Instead, the raw data need to be cleaned and organized into a usable format. Data preprocessing is an essential step used to clean the data and make them useful for any experiment associated with machine learning. Cleaning the data includes replacing or removing missing values, fixing structural errors, and applying discretization for certain continuous variables such as the age of individuals [39]. Before using the data, all the redundant or unnecessary variables must be removed from the database. Null values must also be handled [39].

**Figure 3:**
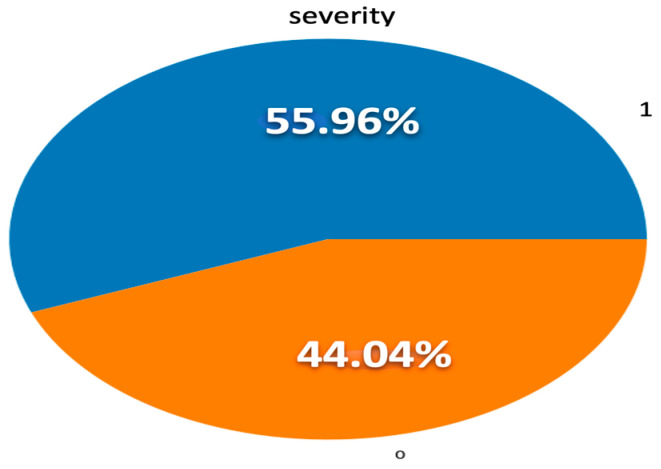
Imbalanced dataset. 1 represents “Severe cases”. 0 represents “Mild cases”.

In this study, data preprocessing was utilized to improve the quality of data, to support us to build an accurate machine learning model [40]. The data preparation and preprocessing steps were implemented using machine learning methods. Data cleaning was performed using Jupyter Notebook in a Google Colab environment, as follows:

- Converting value data of some attributes into the required format based on the model specification, such as converting gender values from the string datatype to the numerical datatype as is widely used in machine learning algorithms [41].
- Removing redundant or unnecessary attributes, i.e., some vital signs with the same meaning, such as “body temperature” and “fever”.
- Replacing and handling missing values either by using imputation methods such as mean, mode, etc., or by coming back to the data source (physicians who shared in the data collection process) to find the value of any missing variable of a specific case in the dataset.
- Checking all features in the dataset had no null values, as no ML method can handle these NULL or Nan values on its own [39].
- Balancing the dataset, which was slightly imbalanced initially, as shown in Figure 3. The class of severe cases was a major class. In this research, SMOTE methods were used to handle the imbalanced dataset [42].

### 3.5. Data Analysis and Feature Selection

Data analysis is the process of systematically applying statistical or logical techniques to describe and illustrate, condense, recap, and evaluate data. It aids in organizing the data to understand the data completely and detect useful information. Also, it aids in enhancing data quality, which is necessary to operate machine learning models efficiently [43].

#### 3.5.1. Exploratory Data Analysis

Exploratory data analysis methods are often called descriptive statistics [44]. They are considered simple ways to obtain a big picture of the data and immediate ways to check for mistakes. They aid in summarizing the raw data, discovering important features, describing the distribution of a single variable (center, min., shape, outliers), checking data for errors, and investigating relationships between features [44]. Table 3 below shows some of the common metrics, such as count, mean, standard deviation (std), and minimum and maximum values.

From the previous table and after investigating the statistics of features, we found that for some features, such as “Diarrhea for 14 days or more”, all values were 0. It meant this feature did not add value to the severity prediction framework and we could remove it from the dataset. Therefore, it was excluded from the dataset before applying ML methods.

#### 3.5.2. Statistical Data Analysis

**Figure 4:**
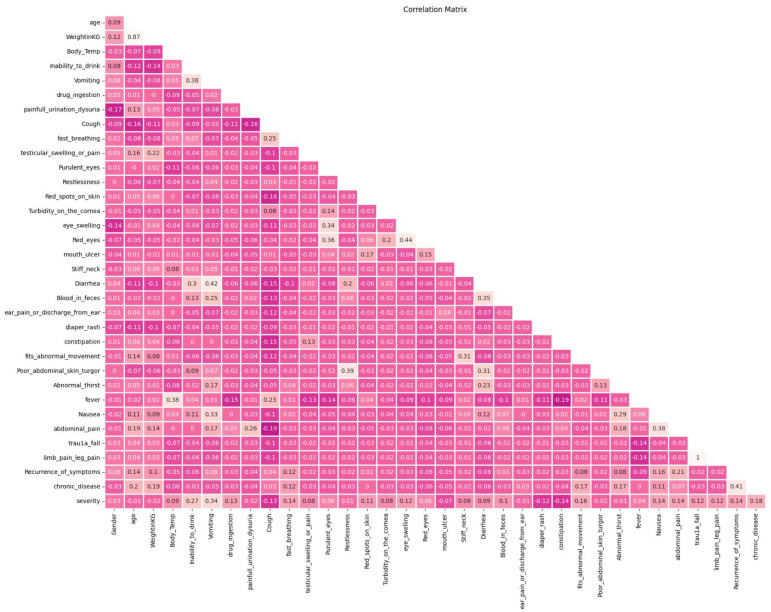
Correlation analysis.

Statistical analysis means investigating trends, patterns, and relationships of dataset features. It is important to uncover patterns and summarize and visualize the data [45], including organization, description, correlations, discovery of the interactions between factors, and interpretation of data [46]. Correlation analysis is a common method of statistical analysis that measures the association between two variables. It is used to measure the strength of the relationship between two variables or features and compute their association [47]. Figure 4 shows the correlation analysis between the features of the data used in building the model: dark color refers to a strong relationship between variables and light color refers to a weak relationship between variables.

After investigating the correlation analysis, the “children’s height” feature was removed from the dataset since it had a very small correlation with the severity of the children’s disease, as shown in Table 4.

There were some other features that had a weak relationship with severity level, as shown in Figure 4. However, we found that it was better to use these features for building the proposed model to achieve more reliable results. That is, the pediatric physicians (data source) mentioned that it was better not to exclude any of these features to avoid missing some of the common severe children’s cases.

#### 3.5.3. Data Visualization

**Figure 5:**
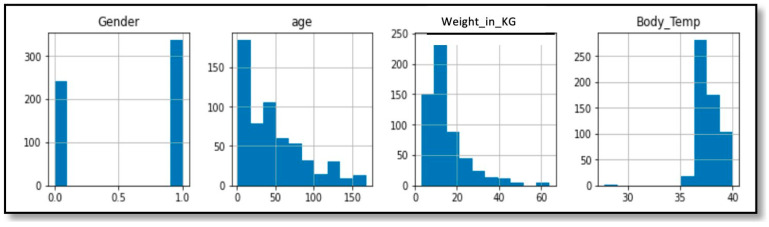
Histograms of child cases’ vital signs.

**Figure 6:**
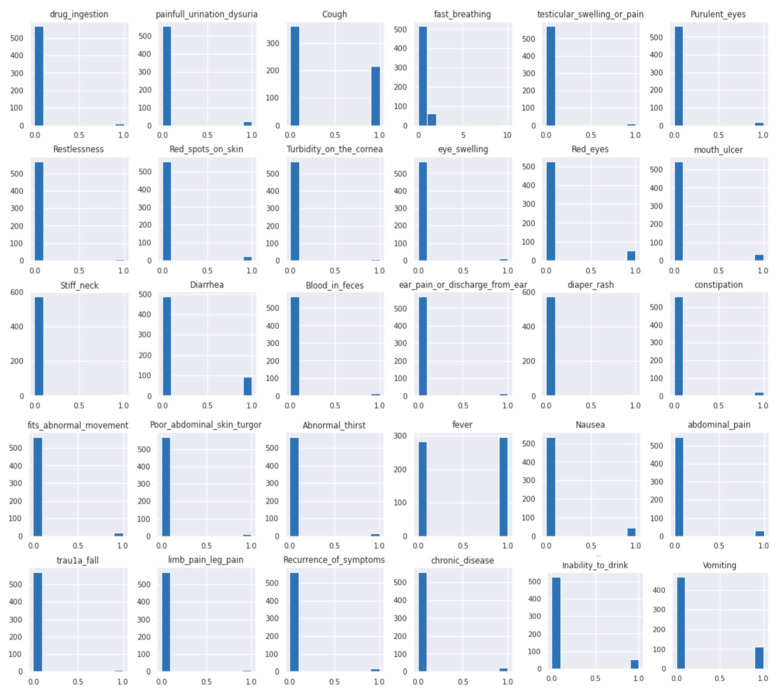
Histograms of child cases’ symptoms.

Histograms are used to summarize the distribution of features in the dataset graphically [48]. The following Figure 5 and Figure 6 show histograms of the features in the dataset. Figure 5 shows how the children’s vital signs were distributed and their range; referring to this helped us to ensure all values of the features were in the right range, such as age. The histogram shows all cases were in the age range between 1 month and 168 months (14 years).

Figure 6 shows histograms of the child cases’ symptoms. It is obvious that coughing, fever, diarrhea, and vomiting were the most common children’s symptoms. Additional symptoms were less common in these children’s cases but usually associated with one of the common symptoms. Therefore, the less common symptoms were necessary to distinguish whether the child’s common symptoms related to severe or mild illness.

### 3.6. Proposed Framework Implementation

**Figure 7:**
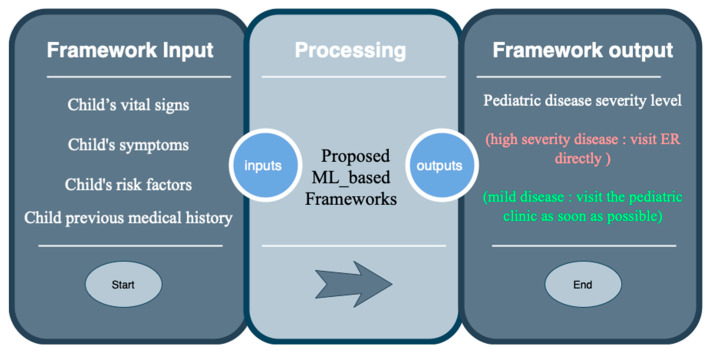
Framework input and output.

The framework for detection of pediatric symptom severity proposed in this study is aimed at distinguishing whether mild, recurring, and common pediatric symptoms relate to mild or severe children’s diseases. The framework inputs are the child’s vital signs, symptoms, risk factors, and previous history. The framework output is the severity level of the children’s case and whether it needs urgent medical intervention to avoid long-term morbidity or death. The proposed framework inputs and outputs are illustrated in Figure 7. The model in the proposed framework is designed to be useable by parents or anyone who does not have a medical background (without visiting a hospital).

The model was created using the Python programming language through the Jupyter Notebook in a Google Colab environment. The model-building process starts with model training to produce a targeted model that can predict output results [49]. The proposed framework was implemented on balanced, cleaned data, as outlined in the previous sections. The dataset was split into two subsets, with the training set comprising 70% of the total dataset and the remaining 30% used as the testing set. The training set is the dataset that can be used to train the model, while the testing dataset can be used to test the model; it should be separated from the training set, but should follow the same probability distribution as the training set so that the testing set can be used to measure the performance of the trained model [50]. In this study, the training dataset was used to create the proposed framework for a pediatric disease severity detection model.

In this study, nine ML methods were applied to build the severity detection model. The methods were decision tree, random forest, AdaBoost, bagging classifier, stacking classifier, XGB, support vector machine, voting, and logistic regression, as these methods are the most widely used in healthcare and several studies in the literature have used them successfully to detect disease severity [19,21,51,52]. These methods also produced an efficient pediatric disease severity detection model in this study. All the machine learning algorithms used in this research were supervised machine learning (SML) methods. An SML method utilizes a training dataset that has predictor variables and labeled data (target variable) to predict results [46]. The following gives a brief description of the ML methods used.

- Decision tree (DT): The DT algorithm builds a classifier model to work hierarchically like a tree structure. Its structure consists of branches and nodes on the basis of evidence collected for each attribute during the model training phase. It tends to work as a set of if–else conditions to visualize the data and classify them according to the conditions to predict the results [16,25]. A decision tree can be used to represent decisions visually and explicitly [28].
- Random forest (RF): The RF algorithm is considered an ensemble learning method, as it builds a model by constructing a multitude of decision trees during training [53]. It starts to generate multiple random trees called a forest. Then, it attempts to combine the trees using an estimated outcome and voting procedure during the prediction process. Merging the random trees by voting in the forest aims to enhance the prediction accuracy for future data [53]. It can be used for both classification and regression. When there is more than one tree in the forest, this may help to achieve results with high accuracy [54]. In this study, the number of trees was set at 500 for building the RF model.
- Bagging: This is one of the most commonly used ensemble learning methods. It is also called bootstrap aggregation. It combines several classifiers using training data, in which different training data are presented for learning in each model. The new training set is generated based on randomly selected examples with replacements from the original training set [42]. In this study, the number of trees per hyperparameter tuning was set to 1000 with the bootstrap method.
- Stacking: This is one of the most popular machine learning ensemble modeling methods. It works by assembling various weak learners in a parallel way that combines them with meta learners [55]. This ensemble approach works by using the combined input of multiple weak learners’ predictions and meta learners so that a better output prediction model can be achieved. The stacking algorithm takes the outputs of sub-models as input and attempts to learn how to best combine the input predictions to make a better output prediction [55].
- Voting: This is a machine learning method that trains the new model based on multiple other models and predicts an output class based on the highest probability of the chosen class as the output. It simply aggregates the findings of each classifier passed into the voting classifier and predicts the output class based on majority voting. It is used to dedicate previous models and trains a new model by using these models to predict the output based on their combined majority of voting for each output class and by finding the accuracy for each of them, instead of creating a single model. It is used to improve model performance, ideally achieving better performance than any single model used in the ensemble [56].
- AdaBoost (adaptive boosting): This is an ensemble method that iteratively trains a series of weak classifiers that are constructed based on weighted data to enhance their efficiency [57]. In the AdaBoost method, weak learning models with better accuracy can be boosted to create a strong prediction model [57].
- Support vector machine (SVM): The SVM is used for both classification and regression challenges. It uses the coordinates of individual observations. It works by plotting each data item as a point in n-dimensional space (n is the feature number of the data), with the value of each feature being the value of a particular coordinate. Then, it performs classification by finding the optimal hyper-plane for differentiating the two classes, where a hyper-plane is a form of SVM visualization [49].
- Logistic regression: This is used to predict the probability of a target variable. A logistic regression model predicts a dependent data variable by analyzing the relationship between existing independent variables [58].
- XGB (extreme gradient boosting): EGB is one of the machine learning tree models. It is an ensemble of decision tree methods where a pruning strategy is applied to correct the errors made by earlier trees. Trees are added to the model until no additional improvement can be noted. It is one of the best machine learning algorithms for its speed and performance and it can handle missing data, skewed class distributions, and a large dataset [59].

### 3.7. Proposed Framework Evaluation

The methods in this framework were evaluated and compared in terms of accuracy, precision, recall, F1 score, and ROC AUC [52], and those with the best fit were selected in the framework for detecting the severity of children’s diseases. These main metrics were valuable for measuring the results of ML prediction. After our evaluation, those with high values were mapped and compared with relevant models from the literature, to conclude which was best for detecting the severity of pediatric diseases. The results of our evaluation and comparison with relevant models from the literature will be discussed in the next section.

## 4. Results

After evaluating the ML methods using the testing set, the best classifiers in terms of accuracy in detecting the severity of mild and recurrent pediatric symptoms were DT and RF. These achieved accuracies of 94.38% and 94.25%, respectively, while the remaining seven gave good accuracies between 85% and 94%, as shown in Table 6.

All ML methods were evaluated in terms of accuracy, precision, sensitivity/recall, F1 score, and ROC AUC score after computing the confusion matrix. These measures are calculated using different equations, as mentioned below.

- Confusion matrix: This is helpful to predict classification problems. It represents the results based on a positive or negative ratio of predicted and actual values. It helps to calculate other metrics of a model using four values: true positive (TP), true negative (TN), false positive (FP), and false negative (FN) [60]
- Accuracy: This is the ratio of correct prediction cases to all entered cases. It measures the total true prediction divided by total predictions. It is calculated using the formula [61]:

Accuracy = TP + TN/TP + TN + FP + FN. (1)

- Precision: This is a classification metric that is used to find items that are incorrectly labeled among the given class. It compares the correctly identified severe cases to all severe cases. The range of precision values is from 0.0 to 1.0 [62]. It is calculated using the formula:

PREC = TP/(TP + FP).(2)

- Recall/sensitivity: This is used to measure the performance of the model. It indicates the missed positive predictions. It is the ratio of correct positive predictions to all positive predictions that could have been made in that class. The range of recall values is 0.0 to 1.0 [62]. It is calculated using the formula:

Recall = TP/(TP + FN)/(TP/P). (3)

- F1 score: This is a weighted average of recall and calculated precision value. It is calculated using the formula [62]:

F1 = 2TP/(2TP + FP + FN). (4)

- ROC AUC: The receiver operating characteristic (ROC) is a graphical curve that measures the performance of classifiers at various thresholds, while the area under the curve (AUC) is a measure of the classifier’s ability to distinguish between classes. It is a summary of the ROC curve, where a higher AUC value means a better performance of the model at distinguishing between the positive and negative classes [42].

A confusion matrix of the selected ML methods is shown in Table 5 below.

**Figure 8:**
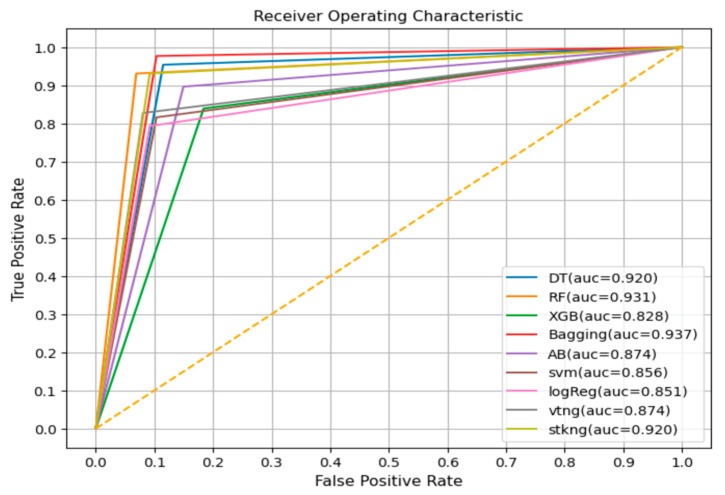
ROC AUC for selected ML methods.

Table 6 reports the outcomes of testing the proposed classifiers for their efficacy to detect the severity of mild pediatric symptoms. Figure 8 shows the ROC and ACU for all the selected ML methods.

The comparison above illustrates that the best ML methods to detect the severity of children’s common, mild, and recurrent symptoms in terms of accuracy were DT, RF, and bagging, with an accuracy of about 94%.

After evaluating the ML methods, those that achieved high measures values were mapped and compared with relevant models from the literature to conclude which was the best for our pediatric disease severity detection model. The results of this comparison are discussed in the next section.

## 5. Discussion

In this section, the proposed model will be compared with related works considering efficiency and usability criteria. The main contribution of the proposed framework is to enable parents, by using this proposed model, to check if their children’s mild symptoms potentially signify severe disease and need urgent medical intervention. To that end, the model is able to detect the emergency of a pediatric disease in the early stage from mild symptoms. In severe cases, the model will detect the emergency of a pediatric disease from mild symptoms; otherwise, if the child has a mild case, it finds there is no need for immediate medical intervention.

Children differ from adults, and some severe diseases occur in children as mild symptoms. If these diseases are not treated in time at an early stage, they will worsen the child’s health rapidly (within a few days) and may cause child morbidity or death.

Parents should be aware of some serious and alarming symptoms. However, with milder symptoms, it is difficult for parents to know if a child’s new symptoms signify dangerous or mild diseases, potentially linked to the educational and socioeconomic levels of parents [4]. Uncertainty might result in the child missing the appropriate chance to go to the hospital, which may cause the child’s mortality or morbidity.

The proposed model can be considered a reliable, useable, and effective model compared with related works from the literature. This is because most of the previous studies investigated in the literature review restricted their models to specific pediatric diseases, which affects their usability. It is illogical that whenever a patient feels new symptoms, the patient should use more than one model to check their condition [63]. It seems that there is a lack of broadly applicable models that are designed to be used by patients. In pediatrics, there is a particular need for models that incorporate children’s recurrent symptoms when detecting the severity of the case, while also considering the patient’s medical history and risk factors [63].

The proposed model has a ranged restricted to a specific group of symptoms and it is clear which group of users it is designed for. In this research field, it is important to understand where and to whom it is reasonable to apply a model and find if they obtain a benefit from it [63]. The scope of this research was defined after conducting interviews with physicians from pediatrics and emergency medicine. They agreed that the early detection of pediatric illness from mild/recurrent symptoms is an important issue in disease management, and they proposed that introducing a model in this area will allow for a better and more rapid cure of illnesses while reducing complications. However, these interviews highlighted common issues when applying ML techniques in medicine, which are the lack of interaction alignment, lack of regulation, and cognitive dissonance between the intuition of the ML architect and health professionals. This study represents a step toward improving attention and cross-fertilization between ML architects and health professionals, to produce a more effective ML model for healthcare purposes [64].

As illustrated previously in the literature review, there is a need to aid parents in determining the severity of their children’s illness and finding out if the there is a serious disease behind mild and recurring symptoms. The literature review showed that there are very few existing systems that detect the severity of children’s symptoms and can be used by parents without visiting the hospital [24]. The proposed framework is designed to aid the parents of a sick child through detecting the disease severity and observing and recording recurring minor symptoms, to predict if they indicate a serious illness. It also considers the risk factors and previous medical history. Regarding efficiency criteria, this research found relatively little information due to the novelty of the subject [65]. Table 7 shows a comparison between the proposed model and some from previous related work. Even though all these studies had bigger datasets than this research and were conducted to cover only one disease, they had very similar accuracy levels to this work.

## 6. Conclusions

Children are considered a vulnerable group in medicine as they are susceptible to sickness, having prolonged health effects, and dying from some diseases. Most of these diseases occur in sick children at an early stage as mild or recurrent symptoms. If these diseases are discovered in time, they can be fully treatable and most of their long-term effects can be fully prevented. However, as mentioned in the literature review, there are very few existing systems that detect the severity of children’s common symptoms and can be used by parents without visiting the hospital. Parents have the difficult job of making a judgment of their children’s health; it is often hard to know what health concerns normal and what symptoms are critical or emergency signals.

This research was conducted to develop a framework usable by parents for detecting the severity of children’s mild and recurrent symptoms. This is to aid in reducing children’s morbidity and mortality from preventable and treatable cases that can be fully cured if they are detected in the early stages. The model is able to detect the severity of current symptoms, as well as taking into consideration the child’s vital signs, risk factors, and previous health history. The main advantage of the proposed framework is that it enables parents to determine whether their children’s seemingly minor symptoms could be signs of a serious illness that requires immediate medical attention.

To accomplish the purpose of this study, nine machine learning methods were trained and compared to identify the optimal prediction model. To achieve the best results for this research goal, data from 579 children’s cases were collected. The essential criteria for choosing the children’s cases were that a child visited the hospital with mild/common symptoms and his/her final diagnosis was either a severe case or mild case.

The dataset was preprocessed, and a class imbalance was handled using the SMOTE method. Additionally, exploratory and statistical data analyses were performed, like correlation analysis, and histograms were generated to ensure that all values of the feature were in the right range, as well as reveal the associations between features, so we could check the data for errors of feature values and investigate relationships between them. The well-preprocessed dataset enhanced the model’s performance and accuracy.

After applying the selected ML methods discussed in the previous sections, we assessed those in terms of accuracy, precision, recall/sensitivity, F1 score, and ROC AUC. The most appropriate and reliable, with high accuracy, were decision tree and random forest. These were stronger compared to the others in the current study and the methods investigated in the literature. The accuracy of DT and RF was 94%.

In the future, the proposed model could be tested on bigger datasets to enhance its efficiency and expand its range to cover more uncommon symptoms, so it can detect more pediatric diseases before they become apparent.

## Figures and Tables

**Table 1:** Summary of the current solutions for common pediatric issues using ML.

Research	Research Purpose	ML Algorithm Used	Main Findings/Limitations
Hwang et al., 2022 [16]	Proposed a model to predict critical illness and hospitalization for children	Random forest	The model predicted children’s critical illness efficiently, but it did not consider most of the mild symptoms and parents cannot use it without visiting the hospital
Pan et al., 2021 [28]	Proposed models to rapidly diagnose Mycoplasma pneumoniae pneumonia in children	LR, DT, GBDT, SVM, and MLP	GBDT had the best performance, with an accuracy of 93.7%, but the model can detect the severity of only one kind of children’s disease
Roquette et al., 2020 [10]	Proposed a model for ER pediatric admission and detection of the severity of diseases	Deep neural network, gradient boosting classifier	It was proposed for use in the hospital and its accuracy was 89%, but the model can cover only some common childhood diseases
Bertsimas et al., 2019 [25]	Proposed a model to determine children with important cases of traumatic brain injury	Classification trees	The model helped to reduce unnecessary action in the ER, without missing important patients, but it can only be used to detect the severity of specific cases
Masino et al., 2019 [26]	Evaluated 6 ML algorithms to identify early cases of sepsis in children	LR, NB, SVM, K-NN, RF, and AdaBoost	The ML algorithms that were used showed no significant differences in accuracy, but different algorithms were proposed for specific cases
Iheme et al., 2017 [24]	Implemented a mobile system to enhance pediatric disease diagnostics	Naïve Bayes and decision tree classifier	The model was proposed for use by health workers for children under six years, and the Naïve Bayes algorithms gave a higher accuracy
Mossotto et al., 2017 [27]	Proposed a model to classify pediatric inflammatory bowel	DT, SVM, and linear discriminant	The model was uncertain in discriminating between some diseases that have common symptoms

**Table 3:** Features’ statistics.

Feature	Count	Mean	Std.	Min.	50%	Max.
**Gender**	579.0	0.583765	0.493360	0.0	0.0	1.0
**Age**	579.0	46.519862	40.257143	1.0	12.0	168.0
**Weight in kg**	579.0	15.204145	9.327476	3.0	9.0	64.0
**Body temp.**	579.0	37.608981	1.045681	36.5	37.0	40.0
**Inability to drink** **or suckle**	579.0	0.093264	0.291054	0.0	0.0	1.0
**Vomiting**	579.0	0.193437	0.395334	0.0	0.0	1.0
**Drug ingestion**	579.0	0.020725	0.142587	0.0	0.0	1.0
**Paling**	579.0	0.039724	0.195478	0.0	0.0	1.0
**Cough**	579.0	0.374784	0.484486	0.0	0.0	1.0
**Ear pain or discharge**	579.0	0.126079	0.515833	0.0	0.0	1.0
**Liquid or soft defeating**	579.0	0.015544	0.123810	0.0	0.0	1.0
**Purulent eyes**	579.0	0.031088	0.173706	0.0	0.0	1.0
**Diarrhea for 14 days or more**	579.0	0.0	0.0	0.0	0.0	0.0
**Restlessness**	579.0	0.017271	0.130393	0.0	0.0	1.0
**Red spots on skin**	579.0	0.039724	0.195478	0.0	0.0	1.0
**Turbidity on the cornea**	579.0	0.015544	0.123810	0.0	0.0	1.0
**Hollowed eyes**	579.0	0.018998	0.136637	0.0	0.0	1.0
**Red eyes**	579.0	0.091537	0.288621	0.0	0.0	1.0
**Mouth ulcer**	579.0	0.060449	0.238523	0.0	0.0	1.0
**Stiff neck**	579.0	0.008636	0.092606	0.0	0.0	1.0
**Diarrhea**	579.0	0.158895	0.365894	0.0	0.0	1.0
**Blood in feces**	579.0	0.025907	0.158994	0.0	0.0	1.0
**Fussiness/irritability**	579.0	0.022453	0.148278	0.0	0.0	1.0
**Diaper rash**	579.0	0.012090	0.109381	0.0	0.0	1.0
**Constipation**	579.0	0.034542	0.182775	0.0	0.0	1.0
**Abnormal thirst**	579.0	0.031088	0.173706	0.0	0.0	1.0
**Poor abdominal skin turgor**	579.0	0.017271	0.130393	0.0	0.0	1.0
**Nausea**	579.0	0.077720	0.267962	0.0	0.0	1.0
**Abdominal pain**	579.0	0.055268	0.228700	0.0	0.0	1.0
**Falling**	579.0	0.017271	0.130393	0.0	0.0	1.0
**Persistence of symptoms**	579.0	0.017271	0.130393	0.0	0.0	1.0
**Recurrence of symptoms**	579.0	0.032815	0.178307	0.0	0.0	1.0
**Chronic disease**	579.0	0.039724	0.195478	0.0	0.0	1.0
**Severity**	579.0	0.559585	0.496866	0.0	0.0	1.0

**Table 4:** Correlation coefficient between height and disease severity.

Feature	Severity	Height (cm)
Severity	1	0.10078747
Height (cm)	0.10078747	1

**Table 5:** Confusion matrix of ML methods.

ML Method	Actual (0)		Actual (1)	
	Predicted (0)	Predicted (1)	Predicted (0)	Predicted (1)
**DT**	81	6	4	83
**RF**	81	6	5	82
**Bagging**	79	8	3	84
**Stacking**	79	8	6	81
**Voting**	79	8	14	73
**AdaBoost**	76	11	12	75
**SVM**	78	9	16	71
**LR**	79	8	17	70
**XGB**	72	15	11	76

Note: 0 = not severe case; 1 = severe case.

**Table 6:** Comparison of selected ML methods.

ML Method	Accuracy	Precision	Sensitivity	F1 Score	ROC AUC
Decision tree	94.38%	0.94	0.94	0.94	0.920
Random forest	94.25%	0.94	0.94	0.94	0.931
Bagging	93.67%	0.94	0.94	0.94	0.937
Stacking	91.57%	0.92	0.92	0.92	0.920
Voting	86.78%	0.87	0.87	0.87	0.874
AdaBoost	87.43%	0.87	0.87	0.87	0.874
SVM	85.63%	0.86	0.86	0.86	0.856
Logistic regression	86.05%	0.86	0.86	0.86	0.851
XGB	84.09%	0.84	0.84	0.84	0.828

**Table 7:** Comparison between the accuracy of the proposed model and some from previous related work.

Research	Research Aim	Accuracy	Sample Size	ML Methods Used
Proposed model	ML model to predict severe children’s disease from common symptoms	94%	579 pediatric cases	Decision tree (DT) and random forest (RF)
Hwang et al., 2022 [16]	ML model to predict critical illness among children’s cases in ER	94%	2,621,710 pediatric cases	Random forest (RF)
Sills et al., 2021 [66]	ML model to predict decisions for pediatric patients with asthma who need hospitalization	AUC was 0.886	9069 pediatric cases	Random forest (RF)
Roquette et al., 2020 [10]	ML model to predict the severity of some common pediatric diseases	89%	499,853 pediatric cases	Gradient-boosting classifier and deep neural network (DNN)

## Data Availability

The corresponding author can provide the dataset used in this work upon request.
